# SARS-CoV-2 IgG Antibodies Seroprevalence and Sera Neutralizing Activity in MEXICO: A National Cross-Sectional Study during 2020

**DOI:** 10.3390/microorganisms9040850

**Published:** 2021-04-15

**Authors:** José Esteban Muñoz-Medina, Concepción Grajales-Muñiz, Angel Gustavo Salas-Lais, Larissa Fernandes-Matano, Constantino López-Macías, Irma Eloísa Monroy-Muñoz, Andrea Santos Coy-Arechavaleta, Iliana Donají Palomec-Nava, Célida Duque-Molina, Ruth Lizzeth Madera-Sandoval, Vanessa Rivero-Arredondo, Joaquín González-Ibarra, Julio Elías Alvarado-Yaah, Teresita Rojas-Mendoza, Clara Esperanza Santacruz-Tinoco, Cesar Raúl González-Bonilla, Víctor Hugo Borja-Aburto

**Affiliations:** 1 División de Laboratorios de Vigilancia e Investigación Epidemiológica, IMSS, 07760 Ciudad de México, Mexico; eban10@hotmail.com (J.E.M.-M.); cest03@gmail.com (C.E.S.-T.); 2 Coordinación de Control Técnico e Insumos, IMSS, 07760 Ciudad de México, Mexico; concepcion.grajales@imss.gob.mx (C.G.-M.); teresita.rojas@imss.gob.mx (T.R.-M.); 3 Estancia Posdoctoral (CONACyT), Laboratorio Central de Epidemiología, IMSS, 02990 Ciudad de México, Mexico; salas_lais@yahoo.com.mx; 4 Escuela Nacional de Ciencias Biológicas, IPN, 11340 Ciudad de México, Mexico; larissamatano@gmail.com; 5 Unidad de Investigación Médica en Inmunoquímica, UMAE Hospital de Especialidades, Centro Médico Nacional “Siglo XXI”, IMSS, 06720 Ciudad de México, Mexico; constantino.lopez@imss.gob.mx (C.L.-M.); ruthmaderas@hotmail.com (R.L.M.-S.); vanessa.rivero.207@gmail.com (V.R.-A.); 6 Laboratorio de Genómica, Departamento de Genética y Genómica Humana, Instituto Nacional de Perinatología “Isidro Espinosa de los Reyes”, 11000 Ciudad de México, Mexico; irmae4901@gmail.com; 7 Laboratorio Central de Epidemiología, IMSS, 02990 Ciudad de México, Mexico; sc_arechavaleta@hotmail.com (A.S.C.-A.); ilianapalomec@gmail.com (I.D.P.-N.); julio.alvaradoy@imss.gob.mx (J.E.A.-Y.); 8 Dirección de Prestaciones Médicas, IMSS, 06600 Ciudad de México, Mexico; celida.duque@imss.gob.mx; 9 Coordinación de Investigación en Salud, 06720 Ciudad de México, Mexico; joaquin.gonzalezi@imss.gob.mx (J.G.-I.); cesar.gonzalezb@imss.gob.mx (C.R.G.-B.); 10 Instituto de Salud para el Bienestar, 01020 Ciudad de México, Mexico

**Keywords:** COVID-19, herd immunity, serology, antibody neutralization, clinical laboratory, blood bank, CLIA, ELISA

## Abstract

Until recently, the incidence of COVID-19 was primarily estimated using molecular diagnostic methods. However, the number of cases is vastly underreported using these methods. Seroprevalence studies estimate cumulative infection incidences and allow monitoring of transmission dynamics, and the presence of neutralizing antibodies in the population. In February 2020, the Mexican Social Security Institute began conducting anonymous unrelated sampling of residual sera from specimens across the country, excluding patients with fever within the previous two weeks and/or patients with an acute respiratory infection. Sampling was carried out weekly and began 17 days before Mexico’s first officially confirmed case. The 24,273 sera obtained were analyzed by chemiluminescent-linked immunosorbent assay (CLIA) IgG S1/S2 and, later, positive cases using this technique were also analyzed to determine the rate of neutralization using the enzyme-linked immunosorbent assay (ELISA). We identified 40 CLIA IgG positive cases before the first official report of SARS-CoV-2 infection in Mexico. The national seroprevalence was 3.5% in February and 33.5% in December. Neutralizing activity among IgG positives patients during overall study period was 86.1%. The extent of the SARS-CoV-2 infection in Mexico is 21 times higher than that reported by molecular techniques. Although the general population is still far from achieving herd immunity, epidemiological indicators should be re-estimated based on serological studies of this type.

## 1. Introduction

Severe acute respiratory syndrome coronavirus 2 (SARS-CoV-2) infection can produce a wide spectrum of symptoms and disease severity, from mild and even asymptomatic cases to potentially fatal severe acute respiratory syndrome [1,2].

According to the World Health Organization (WHO), through 31 December 2020, there were 83,955,204 SARS-CoV-2 infections and 1,820,400 deaths associated with COVID-19. The countries most significantly affected to date are as follows: The United States (20,536,742 cases; 360,415 deaths), India (10,286,329 cases; 149,018 deaths), and Brazil (7,675,973 cases; 194,976 deaths) [3]. In Mexico, the first confirmed case occurred on 27 February 2020, and by 31 December 2020, 1,413,935 infections and 124,897 deaths were confirmed [4].

Since the publication of the first genome, diagnostic techniques based on molecular methods have been developed [5]. These show a high degree of sensitivity, especially quantitative reverse transcription PCR (RT-qPCR), which allows determination of the global incidence of SARS-CoV-2 and has been the basis of epidemiological surveillance during the first ten months of the pandemic. Sentinel surveillance, as established in Mexico, samples a percentage of symptomatic cases that meet the appropriate operational case definition. As such, asymptomatic cases and those who do not seek care, despite presenting symptoms, are excluded from these surveillance tallies. The number of officially reported cases is not claimed to reflect all cases; rather, it represents a set of cases that allow monitoring the behavior of the epidemic but not the totality of the disease burden [6].

Beyond this approach, there are serological techniques based on technologies such as enzyme-linked immunosorbent assay (ELISA), lateral flow, chemiluminescence microparticle immunoassay (CIMA), and chemiluminescent-linked immunosorbent assay (CLIA) that can determine both the presence of IgA, IgM, and IgG antibodies (specifically against the N, S, and receptor-binding domain (RBD) proteins of SARS-CoV-2, respectively), and their neutralizing capacities [7,8].

As recommended by the WHO [9], this study monitored changes in seroprevalence over time, which makes it possible to estimate the immunological level of the population. This information is crucial for anticipating the dynamics of the epidemic, predicting, or establishing areas where the appearance of new waves would be more likely, prioritizing them within vaccination programs and both of which aid adaptation of public health response plans [10].

## 2. Materials and Methods

### 2.1. Study Design

Given the imminent introduction of SARS-CoV-2 to Mexico, beginning on 10 February 2020 (two weeks before the first case was detected), the Mexican Institute of Social Security (IMSS) (which provides health care around 60% of the population in Mexico) began gathering an anonymous unrelated sampling (AHS) of residual patient sera. To maintain a population-based focus, specimens were collected from 34 clinical laboratories (CLs; i.e., in each state’s main hospital) and 34 blood banks (BBs; i.e., in either the hospitals or unique to the respective states), across all 32 of the United Mexican States.

Sampling was carried out weekly for 44 weeks (10 February–08 September; 06 October–31 December 2020). Each CL was asked to provide 20 samples of 1 mL of surplus sera, corresponding to two samples from each of the following age groups: 0–20 years, 21–40 years, 41–60 years, 61–80 years, and 81–100 years.

The samples could come from any outpatient collection, excluding patients with fever within the previous two weeks and/or patients with an acute respiratory infection, because the majority of individuals infected with SARS-CoV-2 develop symptoms within a period of 14 days [11] and patients with an acute respiratory infection share symptoms similar to COVID-19 [12]. This exclusion criterion allows the probability of detection of positive cases to be more comparable among the samples obtained from clinical laboratories, and the general population.

To focus on a more limited age range, the BBs were asked for eight weekly samples, six from each of the following age groups: 21–40 years and 41–60 years. However, unfortunately there is not the desired representativeness for each state according to its population, so when grouping by geographic region there is a greater representativeness of the samples by age group to carry out statistical analyzes. The inclusion criteria were the same as those used in BBs to guarantee donation safety [13].

The samples were deidentified, retaining only information on location, age group, and sex. This protocol was approved by the scientific, ethics, and biosafety committees of the IMSS National Scientific Research Commission (R-785-2020-60).

### 2.2. Sample Shipping, Treatment, and Storage

Samples were sent by participating CLs and BBs to the Central Epidemiology Laboratory (CEL), in triple packaging conditions for category B biological samples, following the International Air Transport Association (IATA) recommendations [14]. When samples were received at the CEL, inspection of their physical state was carried out and their reception temperature recorded, after which they were stored at −80 °C until use. Samples were excluded if any of the following applied: not transported in triple packaging; transported outside the refrigeration temperature range (i.e., 4–8 °C); did not meet the defined volume (i.e., 1 mL); did not meet the criteria for anonymous unrelated sampling (i.e., included data traceable to the patient); were lipemic, hemolyzed, spilled, contaminated, or cloudy; did not correspond to the requested time period (i.e., 10 February–31 December 2020); or, were not the requested type (i.e., serum).

Determination of IgG antibodies against the S1 and S2 antigens of the SARS-CoV-2 virus by CLIA.

Determination of IgG antibodies was made from 200 µL of serum using the LIAISON SARS-CoV-2 S1/S2 IgG kit (DiaSorin, Saluggia, Italy No. 311450), following the manufacturer’s instructions. This protocol was authorized for emergency use by the U.S. Food and Drug Administration and registration by the Federal Commission for Protection from Sanitary Risk (COFEPRIS).

Negative and positive controls were used to validate the results. Cutoff values were as follows: negative <12 AU/mL, indeterminate ≥12 to ≤15 AU/mL, and positive >15 AU/mL. The assays specificity ranged between 97–98.5% [15]. The manufacturer asserts that this kit has no cross-reactivity against other coronaviruses (e.g., Human CoV OC43, Human CoV NL63, Human CoV 229E, Human MERS-CoV).

### 2.3. Determination of Neutralizing Antibodies Against the RBD Antigen of the SARS-CoV-2 Virus by ELISA

Determination of neutralizing activity of sera was carried out using the SARS-CoV-2 Surrogate Virus Neutralization Test ELISA Kit (Genscript, Piscataway, NJ, USA. Catalog No. L00847), which was validated by Tan and colleagues [16], with a sensitivity of 100% and specificity of 99.9%.

The tests were carried out in all IgG positive’s samples, following the manufacturer’s recommendation. Negative and positive controls were used to validate the results, with cutoff values as follows: negative <20% inhibition and positive >20% inhibition.

### 2.4. Statistical Analysis

Descriptive statistics were used to express the seroprevalence values, which were defined as the proportion of the total number of positive cases among the total number of serological samples analyzed per epidemiological week, age group, sex, or region. In all the calculated proportions, the 95.0% confidence intervals (CI) were also obtained. The chi-square test for homogeneity and independence, and Fisher’s exact probability test were used to compare categorical variables. *p* values < 0.05 were considered statistically significant. Student’s *t* and one-factor ANOVA were used to compare continuous variables; Mann–Whitney U and Kruskal–Wallis H were used when data did not meet the criteria of normality or homoscedasticity. Analyses were performed using IBM SPSS Statistics^®^ (version 24.0, Armonk, NY, USA) and graphs were generated with GraphPad Prism^®^ (version 6, San Diego, CA, USA), and Microsoft^®^ Excel^®^ (2010, Redmond, WA, USA).

Results are presented for five age groups (0–20 years, 21–40 years, 41–60 years, 61–80 years, and 81–100 years) and the country’s 32 states are divided into five regions: (one) Central (C): Mexico City, State of Mexico, Morelos, Puebla, Tlaxcala, Hidalgo, and Guerrero; (two) Northeast (NE): Durango, Coahuila, Nuevo León, San Luis Potosí, and Tamaulipas; (three) Northwest (NW): Baja California, Baja California Sur, Chihuahua, Sonora, and Sinaloa; (four) West (W): Aguascalientes, Colima, Guanajuato, Jalisco, Michoacán, Nayarit, Querétaro, and Zacatecas; and (five) Southeast (SE): Veracruz, Chiapas, Oaxaca, Campeche, Quintana Roo, Tabasco, and Yucatán.

### 2.5. Role of the Funding Source

The sponsor of the study had no role in study design, data collection, data analysis, data interpretation, or writing of the report. All authors had full access to all the data in the study and the corresponding author had final responsibility for the decision to submit for publication.

## 3. Results

From February to December 2020, 24,273 serum samples were gathered from across the country. This period allowed assessment of the first and second wave of infections in Mexico. Collection started two weeks before the first confirmed case and ended in the second descending phase of the epidemic curve (Figure S1). 

**Figure 1:**
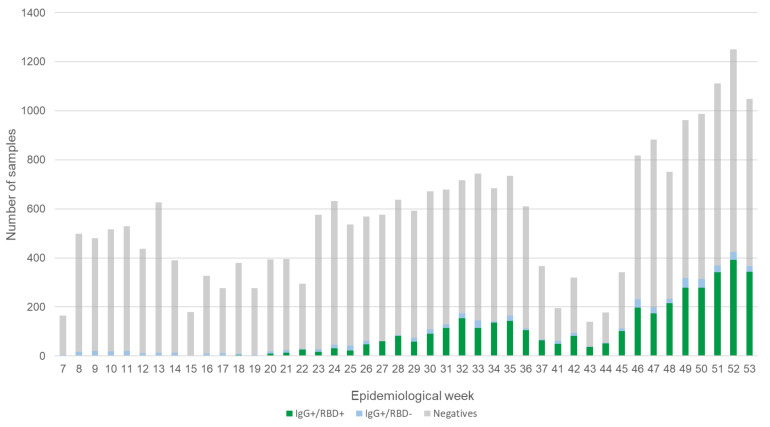
General result of the analyzed samples. The figure shows the total of samples analyzed in each of the weeks included in the study, as well as the results obtained for the IgG detection and neutralization (RBD) assay.

Weekly distribution of all the analyzed samples and their results are presented in Figure 1. Overall, 4488 positive samples for IgG anti S1/S2 were identified; among these, only 86.1% (3863) were also positive for neutralizing antibody detection assay against RBD. On average, seroprevalence reached 33.5% during the previous five weeks (i.e., December 2020).

To identify potential selection biases, the results from both the overall seroprevalences and those for each of the 44 weeks were compared between CLs and BBs. To our surprise, even including criteria to unlink samples from a suspected case, the cumulative seroprevalence differ significantly (CL: 18.9%, CI 18.3–19.5%; BB: 17.7%, CI 16.9–18.5%) (*p* < 0.01). Individual results show differences between BB and CL over several weeks, as shown in Figure S2.

### Dynamics and Extent of Infection (Cumulative Results from 44 Weeks)

**Figure 2:**
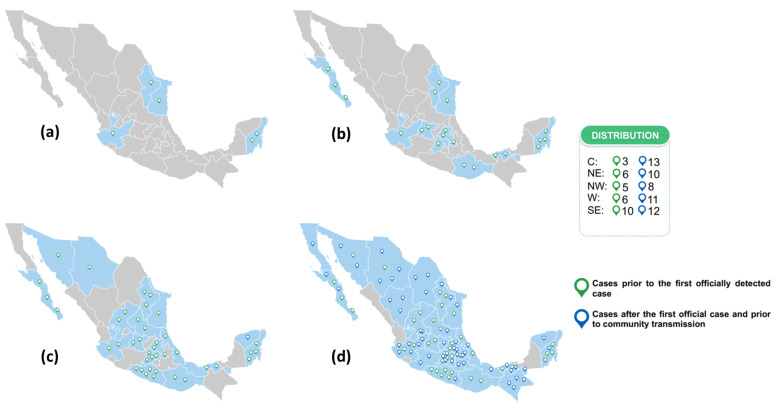
Number of cases detected before the first officially reported case and before community transmission in the country: (**a**) cases detected in epidemiological week 7, (**b**) cases detected until epidemiological week 8, (**c**) cases detected until epidemiological week 9, (**d**) cases detected until epidemiological week 12. Dates considered for the report of cases before the first officially reported: from 02/10/2020 to 02/27/2020 (green symbol). Dates considered for reporting cases before community transmission: until 03/23/2020 (blue symbol).

Since the first week of sampling (epidemiological week 7), positive IgG anti S1/S2 cases of SARS-CoV-2 were identified. From the 1057 samples collected prior to the first case officially detected by the Mexican epidemiological surveillance system on 27 February 2020 (epidemiological week 9), 40 were positive. Another 54 positive samples were detected through the day before community transmission was declared in Mexico (23 March 2020) (Figure 2a–d). Presence of the virus passed from four states (Nuevo Leon, Tamaulipas, Jalisco, and Quintana Roo) in epidemiological week 7 to nearly the entire Mexican territory (29 states) during the next five weeks. All 94 positive results from these samples were confirmed by a second test using the same method (i.e., CLIA).

**Figure 3:**
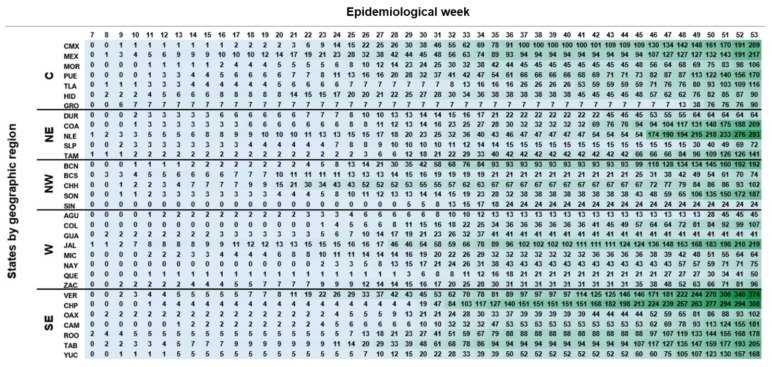
Heat map of accumulated cases by epidemiological week of each state of the Republic. Center region (C): Mexico City (CMX), State of Mexico (MEX), Morelos (MOR), Puebla (PUE), Tlaxcala (TLA), Hidalgo (HID), Guerrero (GRO); Northeast (NE): Durango (DUR), Coahuila (COA), Nuevo León (NLE), San Luis Potosí (SLP), Tamaulipas (TAM); Northwest (NW): Baja California (BCN), Baja California Sur (BCS), Chihuahua (CHH), Sonora (SON), Sinaloa (SIN); West (W): Aguascalientes (AGU), Colima (COL), Guanajuato (GUA), Jalisco (JAL), Michoacán (MIC), Nayarit (NAY), Querétaro (QUE), Zacatecas (ZAC); Southeast (SE): Veracruz (VER), Chiapas (CHP), Oaxaca (OAX), Campeche (CAM), Quintana Roo (ROO), Tabasco (TAB), and Yucatan (YUC). Six of the states (SIN, GRO, NAY, COL, OAX, and QUE) did not comply with the shipment of samples mentioned in Table S1.

The dynamics of infection dissemination across Mexico were evaluated using the accumulated cases within states during the 44 weeks (epidemiological weeks 7–37; 41–53) (Figure 3). The states with the highest number of positive samples during the peak of the first wave in Mexico (epidemiological week 31) were Tabasco, Jalisco, and Baja California. Until the end of August (epidemiological week 35), the states with the most accumulated positive samples by region were State of Mexico (C), Nuevo León (NE), Baja California (NW), Jalisco (W), and Chiapas (SE). During the second wave, the most affected states were Chiapas (CHP), Veracruz (VER), Jalisco (JAL), Nuevo León (NLE), and Coahuila (COA).

**Figure 4:**
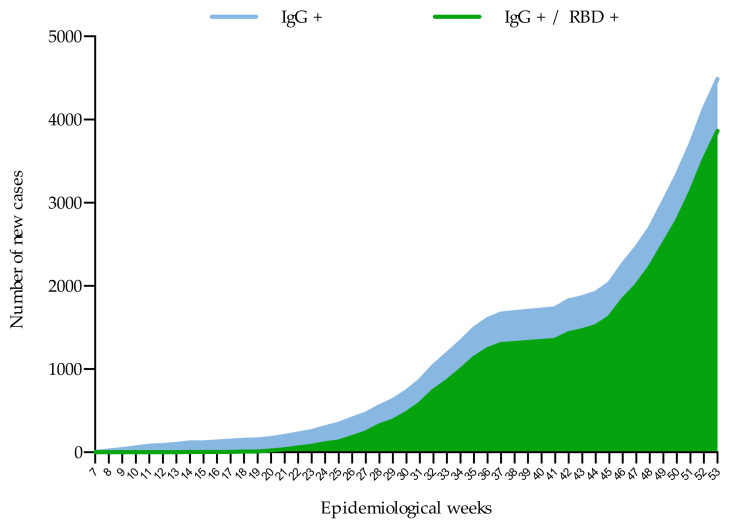
Cumulative frequency of new cases with IgG antibodies to SARS-CoV-2 and neutralizing antibodies (RBD) during the evaluation period (44 weeks).

Although seroprevalence in Mexico increased consistently over time during these weeks of the pandemic, a different pattern was noted with respect to neutralizing antibodies. With few exceptions, the samples with IgG antibodies against S1/S2 did not show neutralizing power until epidemiological week 20 (Figure 4).

**Figure 5:**
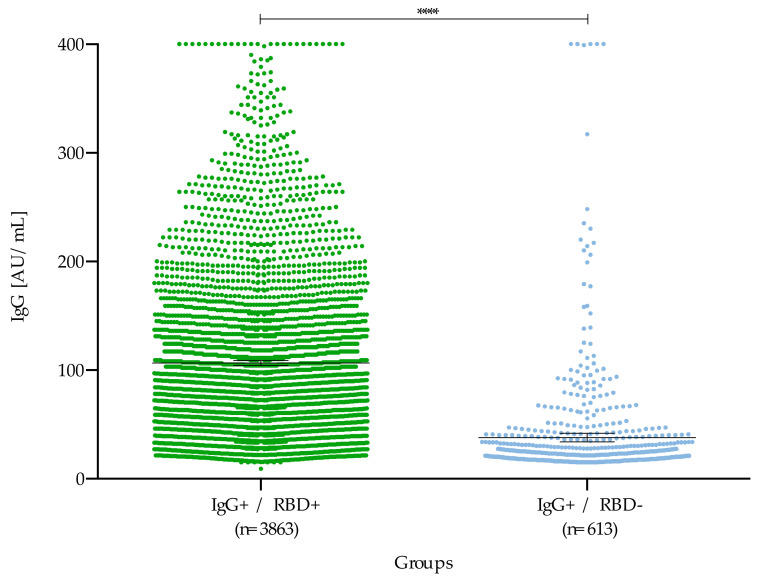
Comparative analysis of double-positive samples (IgG+/RBD+) and that did not show neutralization (IgG+/RBD-). The mean of the groups with neutralizing (IgG+/RBD+) and non-neutralizing (IgG+/RBD-) antibodies was 106.5 AU/mL and 37.7 AU/mL respectively. **** *p* < 0.0001.

When comparing double-positive patients (IgG+/RBD+) with those who were neutralization negative, despite having IgG antibodies (IgG+/RBD–), a higher concentration of IgG antibodies was observed (x = 106.5 AU/mL) for double positives (*p* < 0.05) (Figure 5).

**Figure 6:**
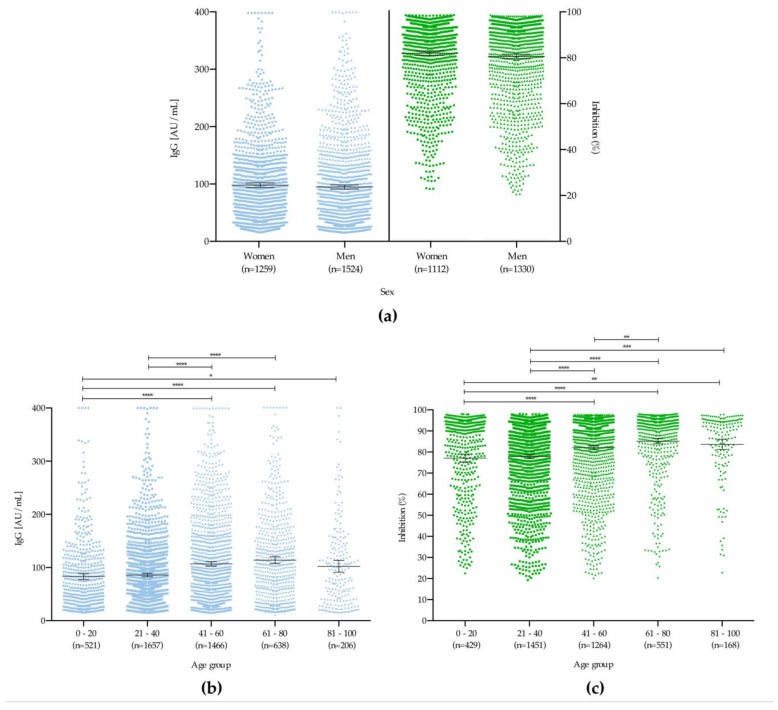
Comparative analysis of IgG concentration and neutralizing capacity against RBD. (**a**) male and female patients and (**b**,**c**): different age groups. * *p* < 0.05, ** *p* < 0.01, *** *p* < 0.001, **** *p* < 0.0001.

Evaluating the production of antibodies between the age groups showed that both the IgG concentration and the rate of neutralization of the antibodies against RBD differed statistically (*p* < 0.05) (Figure 6a), with mean increases with age for both (Figure 6b–c).

**Figure 7:**
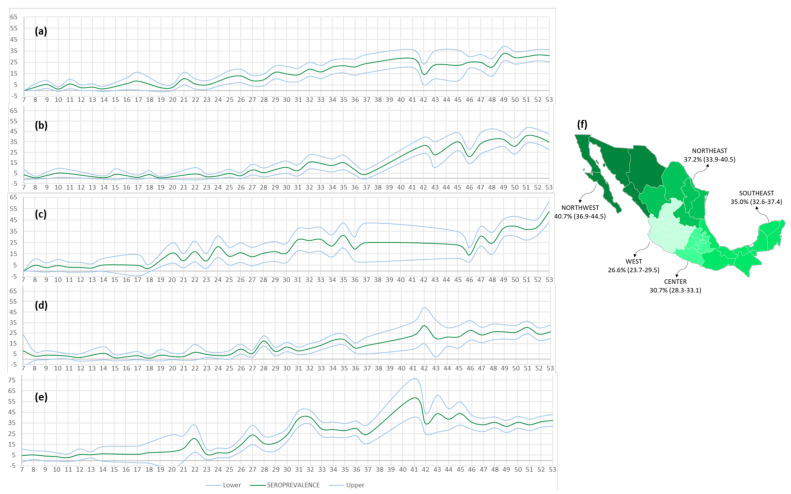
Seroprevalence by region per epidemiological week. The graphs (**a**–**e**) represent the seroprevalence values calculated with their confidence intervals for each of the regions: C, NE, NW, W, and SE, respectively. The map (**f**) shows the December seroprevalence for each region.

The seroprevalence by region and epidemiological week is presented in Figure 7. Furthermore, to present the most current situation in the country, and to avoid dilution of the seroprevalence values from analyzing the cumulative study period, a map of seroprevalence within each geographic region during December alone is presented.

The prevalence of antibodies against SARS-CoV-2 in Mexico during December was 33.5% (CI 32.2–34.7). At that time, NW was the most affected region, with 40.7% (CI 36.9–44.5) of the population already having IgG antibodies against S1/S2, followed by NE (37.2%; CI 33.9–40.5), and SE (35.0%; CI 32.6–37.4). However, through December (the study cutoff date), the C (30.7%; CI 28.3–33.1), and W (26.6%; CI 23.7–29.5) regions appeared least affected and consequently may be the most vulnerable in the future.

The age groups with the lowest percentage of those with the presence of IgG antibodies were 81–100 years (24.3%; CI 19.8–28.8) and those 61–80 years (28%; CI 25.0–31.0). The highest seroprevalence rates were found among the group 21–40 years and 41–60 years, with prevalence rates of 37.7% (35.4–40.0) and 34.0% (31.8–36.2), respectively (Table 1).

For positive samples to IgG antibodies, their neutralizing capacity was also determined by inhibition ELISA. This technique allows for evaluation of the neutralizing capacity in the absence of a biosafety level three laboratory. The results show that adults aged 21–40 years and those aged 41–60 years had the highest percentage of neutralizing antibodies, while old people aged 81–100 years had the lowest prevalence of these antibodies (Table 2).

## 4. Discussion

Over the past year, different strategies have been implemented around the world to mitigate the coronavirus pandemic. Early detection, which allows timely isolation, has been a primary strategy. In this regard, molecular techniques for screening are valuable, as they allow identification of positive cases during the early stages of infection. Other strategies include isolating infected individuals, contact tracing, determining the number of positives, and counting deaths. Serological data are thus essential, as a complement to the regular epidemiological surveillance system, to allow proper estimates of the extent of the infection, the accumulated incidence, and the cases that could be asymptomatic.

Herein, we used two serological techniques, to determine both the extent and dynamics of the SARS-CoV-2 infection in Mexico—using the CLIA technique—and the presence of neutralizing antibodies by ELISA.

Our data show that before the first case was detected by the country’s epidemiological surveillance system, there were already positive cases within the population (Figure 2). The positivity found in February, even considering the limited number of samples analyzed that week and the wide CI, indicates that circulation of SARS-CoV-2 began in Mexico during the last week of January. This is not the first report to identify cases prior to those officially reported [17] and can be explained by considering the operational case definitions used during those months, which restricted confirmatory assessments to those with a travel history to Wuhan, China. Recently, Apolone and colleagues reported the circulation of patients positive to IgG and/or IgM antibodies against SARS-CoV-2 in Italy since September 2019, however, only 5.4% of the antibodies demonstrated neutralizing capacity [18]. While in the US they reported antibodies to SARS-CoV-2 in blood donors in the period December 2019 to January 2020 (106 samples), additionally demonstrating neutralizing capacity against the virus in most of the sera [19].

Unlike other seroprevalence studies recently published [20,21,22], having the opportunity to analyze several weeks prior to the first officially reported case allowed us to observe transmission dynamics. In Mexico, the dissemination of cases was extremely fast (Figure 2), reaching most of the territory in five weeks, which is why community transmission was declared only 25 days after the first reported case [23]. This speed of dissemination and the presence of cases in states that are far removed raise a scenario in which the virus circulated extensively before it was reported, and in which there was more than one introduction to the country. These findings coincide with those reported by Taboada and colleagues, who identified S and G lineages in samples obtained from 27 February to 15 March 2020 [24].

As with transmission dynamics, for seroprevalence at the national level, our study provides a far more time-extensive analysis, making it possible to compare our data with those reported by others. Until March 2021, only two published study had evaluated the seroprevalence of SARS-CoV-2 in Mexico. One of these was carried out in Veracruz City from 1 June to 31 July 2020, with 2174 individuals aged 18 years or older; among the 1141 (52.5%) who were asymptomatic, prevalence was 21.3% [25]. In the present study, samples from Veracruz during the same period and within the same age range yielded lower IgG seropositivity against SARS-CoV-2 (15.2%). The other one was carried out by the National Institute of Public Health in September 2020 [26], whose preliminary results show a positivity of 24.8% for the entire country, a percentage similar to that detected in our study for the same time.

It must be taken into consideration that the seroprevalence calculated in this study, in each epidemiological week, could even be higher, since the quantitative technique used had the limitation of measuring only the IgG antibodies, which means that some samples could still be positive to other type of antibodies, like IgM and/or IgA.

In all the studies carried out in Mexico [25,26], including this one, the results show a considerably higher number of cases, if we compare it with that reported by molecular techniques (15–21 times higher). This indicates a significant underestimation of cases, possibly due to a wide range of factors (e.g., origin characteristics and attributes considered by the surveillance strategy, lack of assistance by health services when initial symptoms appeared, lack of health services in marginalized areas). According to our results, 33.5% of the Mexican population has been exposed to the virus, that is, around 42.5 million Mexicans have generated an antibody response against the virus.

Among the first national-level SARS-CoV-2 antibody seroprevalence studies was that by Havers and colleagues, covering 16,025 residents of 10 cities in the United States (March–May 2020). Their proportion with the presence of antibodies varied from 1.0–6.9%. Compared with our national data during the same period (epidemiological weeks 7–14), 2.9% seroprevalence was registered, with the NW region showing the highest seroprevalence (4.1%). Interestingly, this region includes states that border the United States, indicating probable mobility of Mexicans working in border cities as a cause of viral spread across these areas [27].

Recently, seropositivity to SARS-CoV-2 in the United States through September 2020 varied in the range of <1.0–23.0%, depending on the state, with antibodies against SARS-CoV-2 detected among less than 10.0% of the North American population [28]. Compared with results from the present study during the overlapping sampling month (i.e., August), the average seroprevalence among the Mexican population was higher (21.7%), reaching 27.0% and 32.2% in the NW and SE regions, respectively.

Although the factors that may confer protection remain unknown, if we extrapolate from other viral infections, neutralizing antibodies could correlate with the development of immunity; were this the case, 30.6% of Mexicans would have developed immunity in December 2020. It has been estimated that to achieve group immunity in the case of SARS-CoV-2 infection, 60.0–70.0% of the population is required to be immune [29,30]. Thus, establishment of group immunity in Mexico is still far off.

Based on these new data, the mortality rate in the country should be reviewed and recalculated, since, with official data, the mortality rate in Mexico is one of the highest in the world (10.5%) [4]. Likewise, based on the demonstrated case underestimation, it would also be prudent to recalculate the country’s disease burden, which appears to be much higher than the values currently reported.

Unlike the findings by Bajema and colleagues [28], the highest percentages of seroprevalence were not concentrated exclusively in major urban areas, but also in some states where the population density is low, and visitor numbers are low (e.g., Tabasco). This finding may serve to emphasize prevention strategies in these regions.

Though no significant sex variation was observed in seroprevalence, variation was noted between age groups. The portion of the population that has been most exposed is those who are most economically active (i.e., 21–60 years) (Table 1 and Table 2). This may be related to initiatives taken to protect groups who are presumed to be more vulnerable (i.e., children and older adults). Thus, this may also confirm the success of approaches such as suspending children’s academic activities, as well as isolation of older adults at home. These results may also help guide future vaccination campaigns by prioritizing at-risk groups.

Herein, we have identified that formation of neutralizing antibodies is related to the total amount of IgG antibodies and age, as described by Premkumar and colleagues and Weisberg and colleagues, respectively [31,32]. As shown in Figure 6 and Figure 7, both the percentage of double-positive individuals (IgG+/RBD+) were higher among the group aged 61–80 years. This may suggest the potential for a differential protective response to future vaccines; studies of this type will thus also be needed during the mass immunization period, to verify whether this finding is related to an individual’s immunological maturity or to disease severity, as has been observed in other studies [33].

The present work was limited insofar as our samples were remnants from CLs and BBs, rather than population-based sampling. However, the strength of this study is that it offers a dynamic, temporal view of the pandemic’s spread in Mexico. This initial report is being made within the context of the ongoing pandemic; we will continue both sample collection and seroprevalence analyses in the coming months.

## 5. Conclusions

The actual extent of SARS-CoV-2 infection in Mexico has been far greater than that reported by molecular techniques. Though we are still far from achieving group protection, calculations of all epidemiological indicators of disease burden and mortality should be recalculated based on serological studies of this type.

## Figures and Tables

**Table 1:** Seroprevalence of IgG antibodies (December).

Region	Age Group											
	Total		0–20 Years		21–40 Years		41–60 Years		61–80 Years		81–100 Years	
	Pos/Total	% (CI)	Pos/Total	% (CI)	Pos/Total	% (CI)	Pos/Total	% (CI)	Pos/Total	% (CI)	Pos/Total	% (CI)
C	438/1428	30.7 (28.3–33.1)	43/163	26.4 (19.6–33.1)	166/430	38.6 (34.0–43.2)	152/500	30.4 (26.4–34.4)	61/248	24.6 (19.2–30.0)	16/87	18.4 (10.3–26.5)
NE	314/844	37.2 (33.9–40.5)	41/121	33.9 (25.5–42.3)	98/254	38.6 (32.6–44.6)	105/263	39.9 (34.0–45.8)	48/143	33.6 (25.8–41.3)	22/63	34.9 (23.1–46.7)
NW	267/656	40.7 (36.9–44.5)	29/59	49.2 (36.4–61.9)	92/229	40.2 (33.8–46.5)	97/228	42.5 (36.1–49.0)	37/107	34.6 (25.6–43.6)	12/33	36.4 (20.0–52.8)
W	240/902	26.6 (23.7–29.5)	35/128	27.3 (19.6–35.1)	91/306	29.7 (24.6–34.9)	82/271	30.3 (24.8–35.7)	27/139	19.4 (12.8–26.0)	5/58	8.6 (1.4–15.8)
SE	535/1529	35.0 (32.6–37.4)	77/213	36.2 (29.7–42.6)	216/540	40.0 (35.9–44.1)	145/447	32.4 (28.1–36.8)	67/220	30.5 (24.4–36.5)	30/109	27.5 (19.1–35.9)
Total	1794/5359	33.5 (32.2–34.7)	225/684	32.9 (29.4–36.4)	663/1759	37.7 (35.4–40.0)	581/1709	34.0 (31.8–36.2)	240/857	28.0 (25.0–31.0)	85/350	24.3 (19.8–28.8)

Samples with a positive result (pos); Total of analized samples (total); confidence interval (CI).

**Table 2:** Prevalence of neutralizing antibodies (December).

Region	Age Group											
	Total		0–20 Years		21–40 Years		41–60 Years		61–80 Years		81–100 Years	
	Pos/Total	% (CI)	Pos/Total	% (CI)	Pos/Total	% (CI)	Pos/Total	% (CI)	Pos/Total	% (CI)	Pos/Total	% (CI)
C	401/1428	28.1 (25.8–30.4)	40/163	24.5 (17.9–31.1)	152/430	35.3 (30.8–39.9)	139/500	27.8 (23.9–31.7)	57/248	23.0 (17.7–28.2)	13/86	15.1 (7.5–22.7)
NE	292/844	34.6 (31.4–37.9)	35/121	28.9 (20.8–37.0)	92/254	36.2 (30.3–42.1)	100/262	38.2 (32.3–44.1)	47/143	32.9 (25.2–40.6)	18/63	28.6 (17.4–39.7)
NW	243/656	37.0 (32.6–40.0)	26/59	44.1 (31.4–56.7)	81/226	35.8 (29.6–42.1)	86/225	38.2 (31.9–44.6)	33/107	30.8 (22.1–39.6)	10/33	30.3 (14.6–46.0)
W	211/902	23.4 (20.6–26.2)	32/128	25.0 (17.5–32.5)	81/306	26.5 (21.5–31.4)	72/271	26.6 (21.3–31.8)	21/139	15.1 (9.2–21.1)	5/58	8.6 (1.4–15.8)
SE	494/1529	32.3 (30.0–34.7)	70/212	33.0 (26.7–39.3)	203/540	37.6 (33.5–41.7)	131/446	29.4 (25.1–33.6)	64/220	29.1 (23.1–35.1)	26/109	23.9 (15.9–31.9)
Total	1641/5359	30.6 (29.3–31.8)	203/683	29.7 (26.3–33.1)	609/1756	34.7 (32.5–36.9)	528/1704	31.1 (28.8–33.2)	222/857	25.9 (23.0–28.8)	72/349	20.6 (16.4–24.9)

## Supplementary Materials

The following are available online at https://www.mdpi.com/article/10.3390/microorganisms9040850/s1, Figure S1: Periods used for sampling, Figure S2: Comparison of the results from both the overall seroprevalences and those for each of the 44 weeks were compared between CLs and BBs.
