# Supplementary material for: SARS-CoV-2 IgG Antibodies Seroprevalence and Sera Neutralizing Activity in MEXICO: A National Cross-Sectional Study during 2020
Source: Microorganisms. 2021 Apr 15;9(4):850. doi: 10.3390/microorganisms9040850 (PMC8071542; doi:10.3390/microorganisms9040850)
Supplement: Supplementary file 1 [file microorganisms-09-00850-s001.zip › Supplementary/Figure S1.pdf]

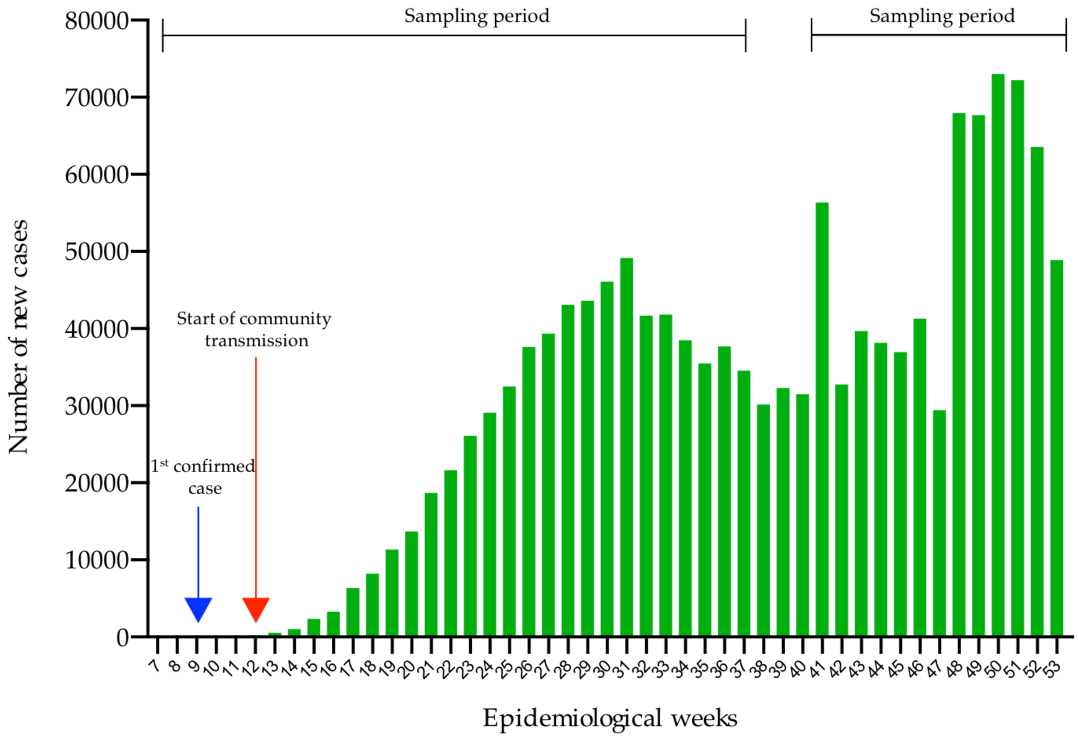

Figure S1. Periods used for sampling. Accessed 19/03/2021, from: https://www.gob.mx/cms/uploads/attachment/file/603816/Comunicado_Tecnico_Diario_COVID-19_2020.12.31.pdf.
