# Supplementary material for: SARS-CoV-2 IgG Antibodies Seroprevalence and Sera Neutralizing Activity in MEXICO: A National Cross-Sectional Study during 2020
Source: Microorganisms. 2021 Apr 15;9(4):850. doi: 10.3390/microorganisms9040850 (PMC8071542; doi:10.3390/microorganisms9040850)
Supplement: Supplementary file 1 [file microorganisms-09-00850-s001.zip › Supplementary/Figure S2.pdf]

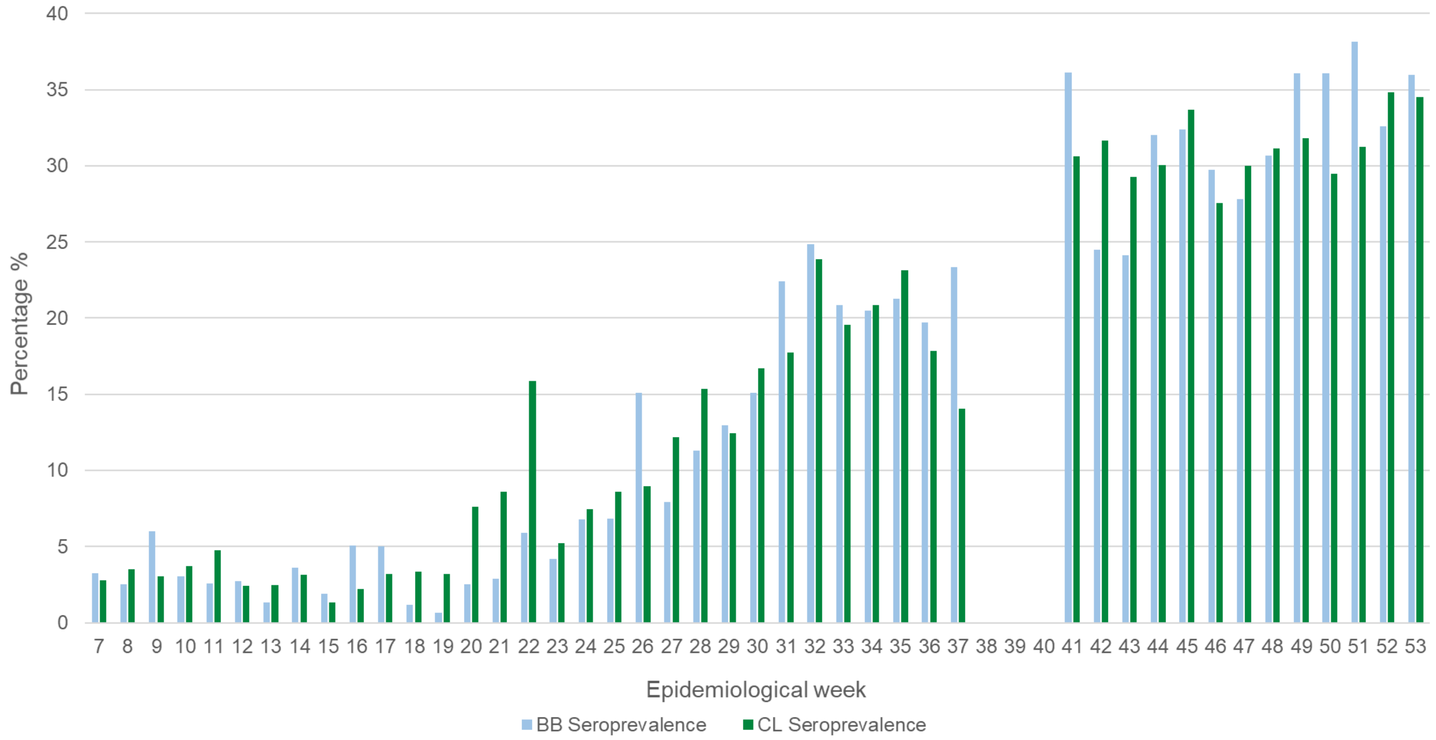

Figure S2. Comparison of the results from both the overall seroprevalences and those for each of the 44 weeks were compared between CLs and BBs.
