# Supplementary material for: SARS-CoV-2 IgG Antibodies Seroprevalence and Sera Neutralizing Activity in MEXICO: A National Cross-Sectional Study during 2020
Source: Microorganisms. 2021 Apr 15;9(4):850. doi: 10.3390/microorganisms9040850 (PMC8071542; doi:10.3390/microorganisms9040850)
Supplement: Supplementary file 1 [file microorganisms-09-00850-s001.zip › Supplementary/Table S1.pdf]

Table S1. Sampling scheme during the period under study.

Clinical laboratory samples						
Age group (years)	0-20	21-40	41-60	61-80	81-100	Total
Number of samples	4	4	4	4	4	20
Volume required	1mL					
Shipping frequency	Weekly					
Sampling period	From February 10 to December 31, 2020					

Blood bank samples			
Age group (years)	20-40	41-60	Total
Number of samples	6	6	12
Volume required	1mL		
Shipping frequency	Weekly		
Sampling period	From February 10 to December 31, 2020		
		Total per week by state	32
